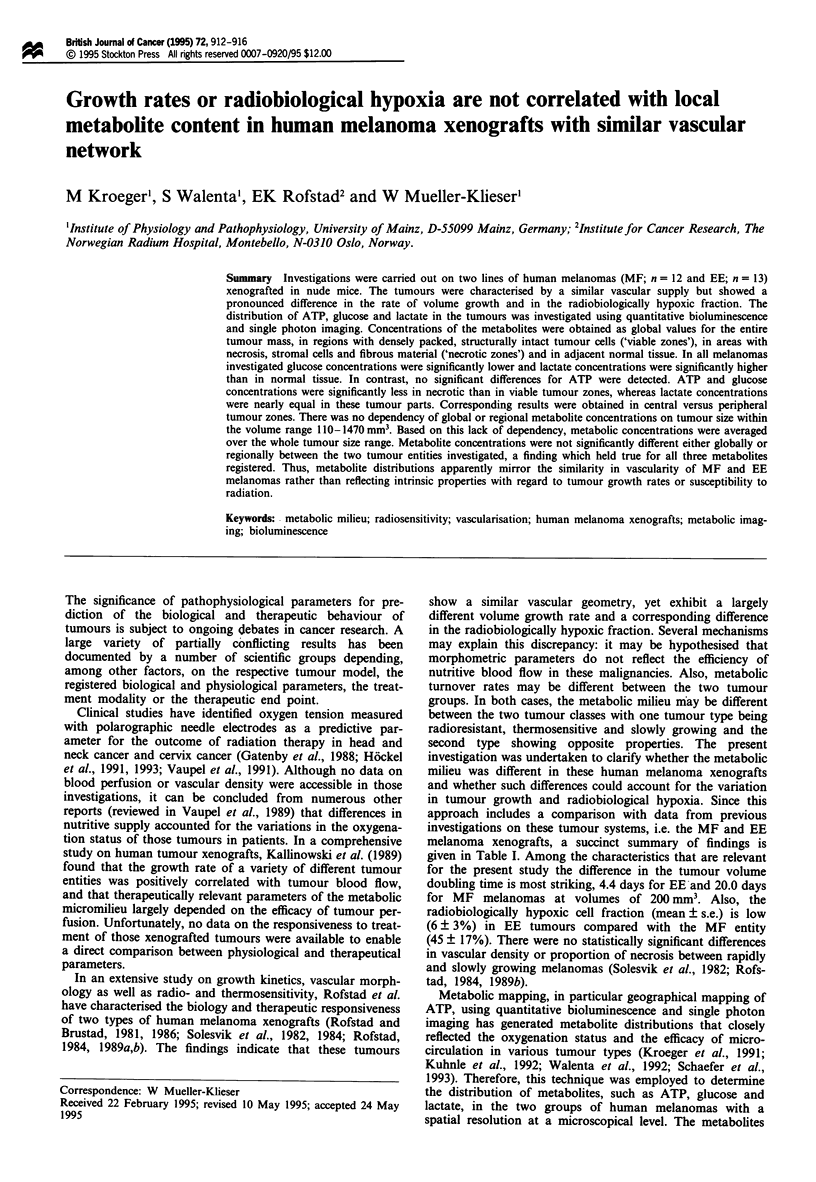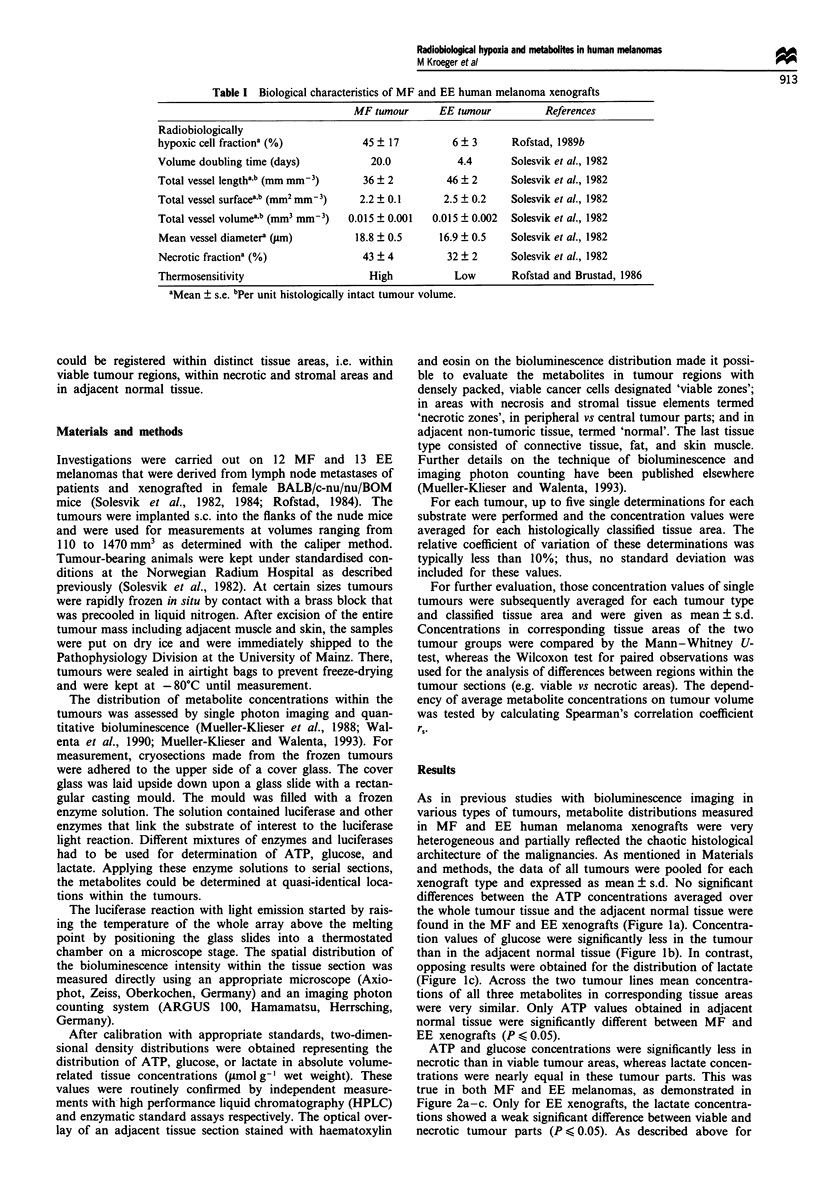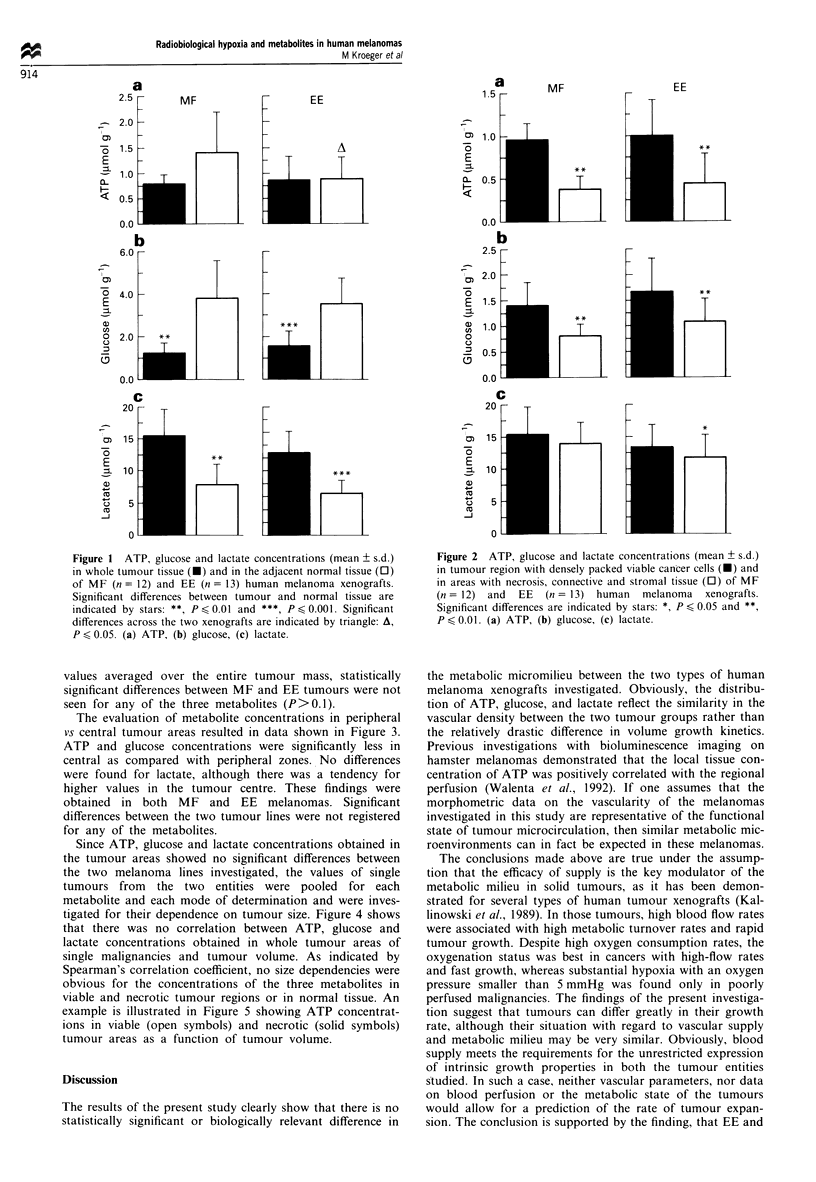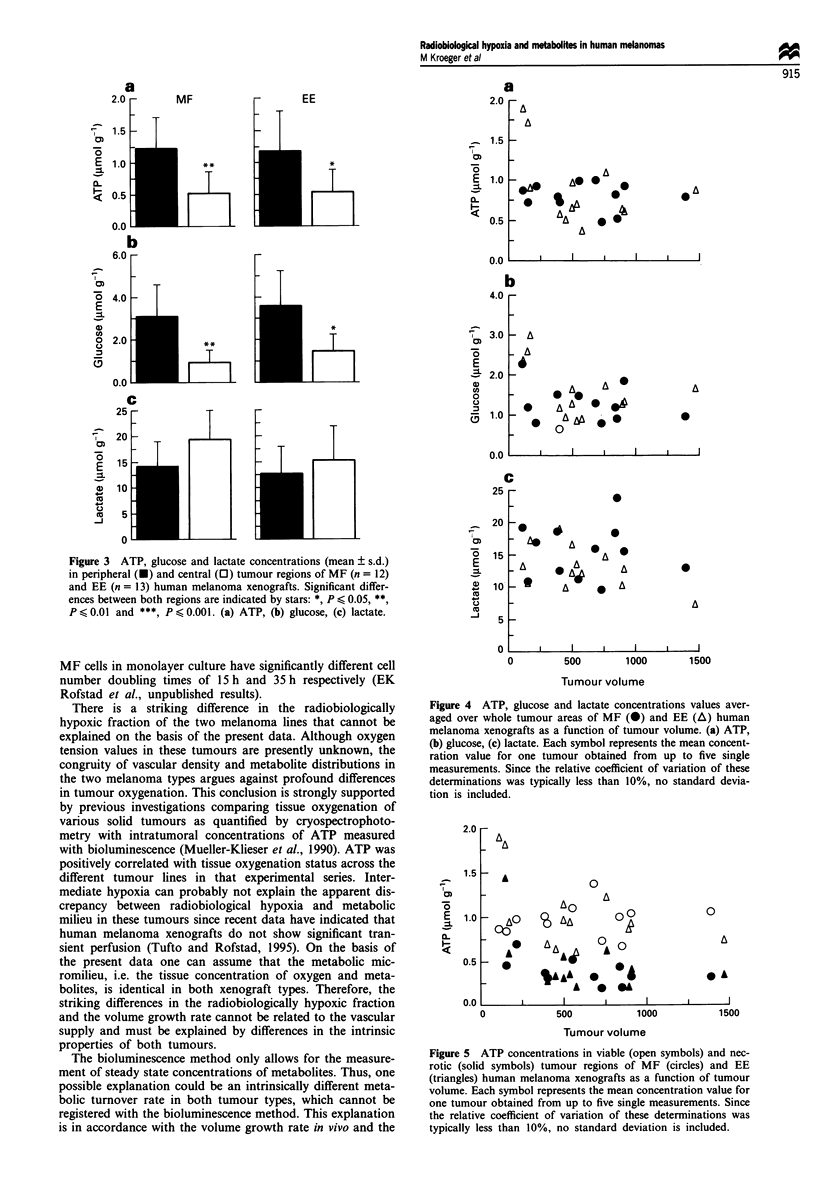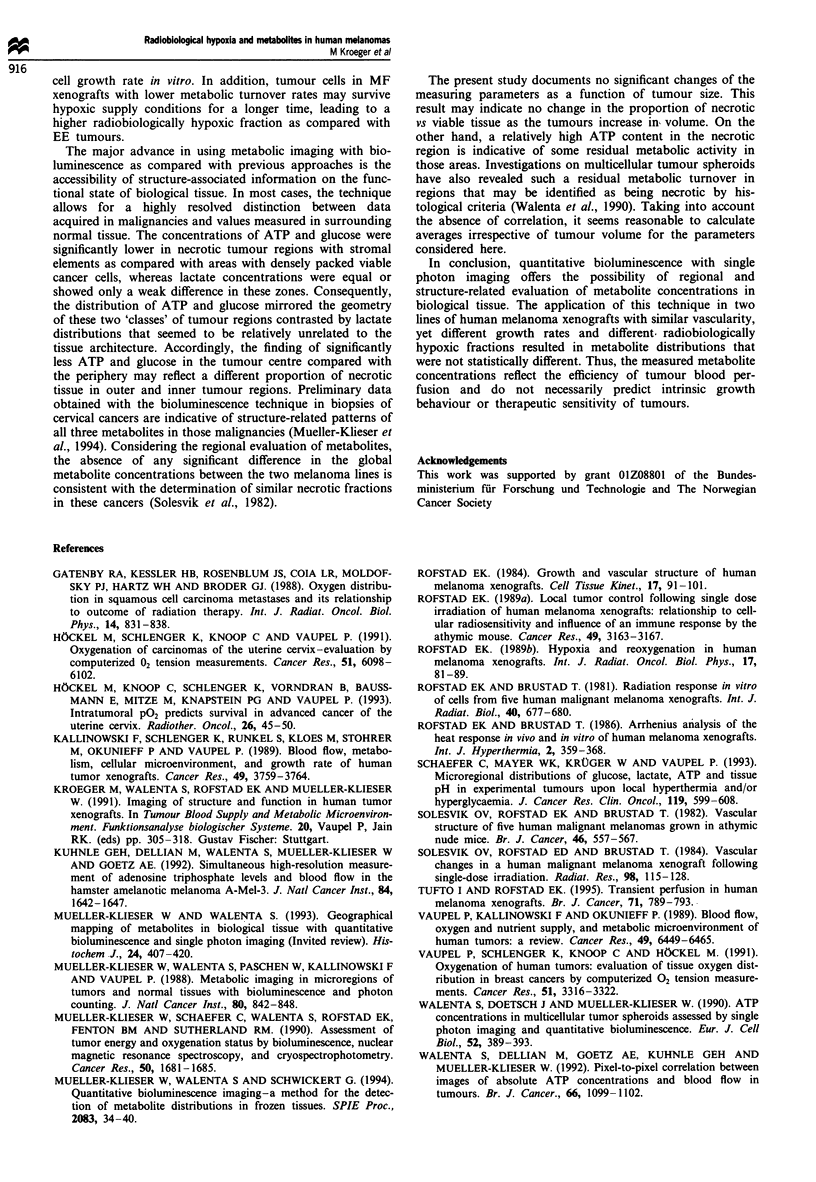# Growth rates or radiobiological hypoxia are not correlated with local metabolite content in human melanoma xenografts with similar vascular network.

**DOI:** 10.1038/bjc.1995.432

**Published:** 1995-10

**Authors:** M. Kroeger, S. Walenta, E. K. Rofstad, W. Mueller-Klieser

**Affiliations:** Institute of Physiology and Pathophysiology, University of Mainz, Germany.

## Abstract

Investigations were carried out on two lines of human melanomas (MF; n = 12 and EE; n = 13) xenografted in nude mice. The tumours were characterised by a similar vascular supply but showed a pronounced difference in the rate of volume growth and in the radiobiologically hypoxic fraction. The distribution of ATP, glucose and lactate in the tumours was investigated using quantitative bioluminescence and single photon imaging. Concentrations of the metabolites were obtained as global values for the entire tumour mass, in regions with densely packed, structurally intact tumour cells ('viable zones'), in areas with necrosis, stromal cells and fibrous material ('necrotic zones') and in adjacent normal tissue. In all melanomas investigated glucose concentrations were significantly lower and lactate concentrations were significantly higher than in normal tissue. In contrast, no significant differences for ATP were detected. ATP and glucose concentrations were significantly less in necrotic than in viable tumour zones, whereas lactate concentrations were nearly equal in these tumour parts. Corresponding results were obtained in central versus peripheral tumour zones. There was no dependency of global or regional metabolite concentrations on tumour size within the volume range 110-1470 mm3. Based on this lack of dependency, metabolic concentrations were averaged over the whole tumour size range. Metabolite concentrations were not significantly different either globally or regionally between the two tumour entities investigated, a finding which held true for all three metabolites registered. Thus, metabolite distributions apparently mirror the similarity in vascularity of MF and EE melanomas rather than reflecting intrinsic properties with regard to tumour growth rates or susceptibility to radiation.


British Journal of Cancer (1995) 72, 912-916
© 1995 Stockton Press All rights reserved 0007-0920/95 $12.00

### Growth rates or radiobiological hypoxia are not correlated with local metabolite content in human melanoma xenografts with similar vascular network

M Kroeger[1], S Walenta[1], EK Rofstad[2] and W Mueller-Klieser[1]

[1]*Institute of Physiology and Pathophysiology, University of Mainz, D-55099 Mainz, Germany;* [2]*Institute for Cancer Research, The Norwegian Radium Hospital, Montebello, N-0310 Oslo, Norway.*

**Summary** Investigations were carried out on two lines of human melanomas (MF; $n = 12$ and EE; $n = 13$) xenografted in nude mice. The tumours were characterised by a similar vascular supply but showed a pronounced difference in the rate of volume growth and in the radiobiologically hypoxic fraction. The distribution of ATP, glucose and lactate in the tumours was investigated using quantitative bioluminescence and single photon imaging. Concentrations of the metabolites were obtained as global values for the entire tumour mass, in regions with densely packed, structurally intact tumour cells ('viable zones'), in areas with necrosis, stromal cells and fibrous material ('necrotic zones') and in adjacent normal tissue. In all melanomas investigated glucose concentrations were significantly lower and lactate concentrations were significantly higher than in normal tissue. In contrast, no significant differences for ATP were detected. ATP and glucose concentrations were significantly less in necrotic than in viable tumour zones, whereas lactate concentrations were nearly equal in these tumour parts. Corresponding results were obtained in central versus peripheral tumour zones. There was no dependency of global or regional metabolite concentrations on tumour size within the volume range 110-1470 $mm^3$. Based on this lack of dependency, metabolic concentrations were averaged over the whole tumour size range. Metabolite concentrations were not significantly different either globally or regionally between the two tumour entities investigated, a finding which held true for all three metabolites registered. Thus, metabolite distributions apparently mirror the similarity in vascularity of MF and EE melanomas rather than reflecting intrinsic properties with regard to tumour growth rates or susceptibility to radiation.

**Keywords:** metabolic milieu; radiosensitivity; vascularisation; human melanoma xenografts; metabolic imaging; bioluminescence

The significance of pathophysiological parameters for prediction of the biological and therapeutic behaviour of tumours is subject to ongoing debates in cancer research. A large variety of partially conflicting results has been documented by a number of scientific groups depending, among other factors, on the respective tumour model, the registered biological and physiological parameters, the treatment modality or the therapeutic end point.

Clinical studies have identified oxygen tension measured with polarographic needle electrodes as a predictive parameter for the outcome of radiation therapy in head and neck cancer and cervix cancer (Gatenby *et al.*, 1988; Höckel *et al.*, 1991, 1993; Vaupel *et al.*, 1991). Although no data on blood perfusion or vascular density were accessible in those investigations, it can be concluded from numerous other reports (reviewed in Vaupel *et al.*, 1989) that differences in nutritive supply accounted for the variations in the oxygenation status of those tumours in patients. In a comprehensive study on human tumour xenografts, Kallinowski *et al.* (1989) found that the growth rate of a variety of different tumour entities was positively correlated with tumour blood flow, and that therapeutically relevant parameters of the metabolic micromilieu largely depended on the efficacy of tumour perfusion. Unfortunately, no data on the responsiveness to treatment of those xenografted tumours were available to enable a direct comparison between physiological and therapeutical parameters.

In an extensive study on growth kinetics, vascular morphology as well as radio- and thermosensitivity, Rofstad *et al.* have characterised the biology and therapeutic responsiveness of two types of human melanoma xenografts (Rofstad and Brustad, 1981, 1986; Solesvik *et al.*, 1982, 1984; Rofstad, 1984, 1989*a*,*b*). The findings indicate that these tumours show a similar vascular geometry, yet exhibit a largely different volume growth rate and a corresponding difference in the radiobiologically hypoxic fraction. Several mechanisms may explain this discrepancy: it may be hypothesised that morphometric parameters do not reflect the efficiency of nutritive blood flow in these malignancies. Also, metabolic turnover rates may be different between the two tumour groups. In both cases, the metabolic milieu may be different between the two tumour classes with one tumour type being radioresistant, thermosensitive and slowly growing and the second type showing opposite properties. The present investigation was undertaken to clarify whether the metabolic milieu was different in these human melanoma xenografts and whether such differences could account for the variation in tumour growth and radiobiological hypoxia. Since this approach includes a comparison with data from previous investigations on these tumour systems, i.e. the MF and EE melanoma xenografts, a succinct summary of findings is given in Table I. Among the characteristics that are relevant for the present study the difference in the tumour volume doubling time is most striking, 4.4 days for EE and 20.0 days for MF melanomas at volumes of 200 $mm^3$. Also, the radiobiologically hypoxic cell fraction (mean ± s.e.) is low (6 ± 3%) in EE tumours compared with the MF entity (45 ± 17%). There were no statistically significant differences in vascular density or proportion of necrosis between rapidly and slowly growing melanomas (Solesvik *et al.*, 1982; Rofstad, 1984, 1989*b*).

Metabolic mapping, in particular geographical mapping of ATP, using quantitative bioluminescence and single photon imaging has generated metabolite distributions that closely reflected the oxygenation status and the efficacy of microcirculation in various tumour types (Kroeger *et al.*, 1991; Kuhnle *et al.*, 1992; Walenta *et al.*, 1992; Schaefer *et al.*, 1993). Therefore, this technique was employed to determine the distribution of metabolites, such as ATP, glucose and lactate, in the two groups of human melanomas with a spatial resolution at a microscopical level. The metabolites

Correspondence: W Mueller-Klieser
Received 22 February 1995; revised 10 May 1995; accepted 24 May 1995

Table I Biological characteristics of MF and EE human melanoma xenografts

| | *MF tumour* | *EE tumour* | *References* |
|---|---|---|---|
| Radiobiologically hypoxic cell fraction[a] (%) | 45 ± 17 | 6 ± 3 | Rofstad, 1989*b* |
| Volume doubling time (days) | 20.0 | 4.4 | Solesvik *et al.*, 1982 |
| Total vessel length[a,b] ($mm\ mm^{-3}$) | 36 ± 2 | 46 ± 2 | Solesvik *et al.*, 1982 |
| Total vessel surface[a,b] ($mm^2\ mm^{-3}$) | 2.2 ± 0.1 | 2.5 ± 0.2 | Solesvik *et al.*, 1982 |
| Total vessel volume[a,b] ($mm^3\ mm^{-3}$) | 0.015 ± 0.001 | 0.015 ± 0.002 | Solesvik *et al.*, 1982 |
| Mean vessel diameter[a] (μm) | 18.8 ± 0.5 | 16.9 ± 0.5 | Solesvik *et al.*, 1982 |
| Necrotic fraction[a] (%) | 43 ± 4 | 32 ± 2 | Solesvik *et al.*, 1982 |
| Thermosensitivity | High | Low | Rofstad and Brustad, 1986 |

[a]Mean ± s.e. [b]Per unit histologically intact tumour volume.

could be registered within distinct tissue areas, i.e. within viable tumour regions, within necrotic and stromal areas and in adjacent normal tissue.

### Materials and methods

Investigations were carried out on 12 MF and 13 EE melanomas that were derived from lymph node metastases of patients and xenografted in female BALB/c-nu/nu/BOM mice (Solesvik *et al.*, 1982, 1984; Rofstad, 1984). The tumours were implanted s.c. into the flanks of the nude mice and were used for measurements at volumes ranging from 110 to 1470 $mm^3$ as determined with the caliper method. Tumour-bearing animals were kept under standardised conditions at the Norwegian Radium Hospital as described previously (Solesvik *et al.*, 1982). At certain sizes tumours were rapidly frozen *in situ* by contact with a brass block that was precooled in liquid nitrogen. After excision of the entire tumour mass including adjacent muscle and skin, the samples were put on dry ice and were immediately shipped to the Pathophysiology Division at the University of Mainz. There, tumours were sealed in airtight bags to prevent freeze-drying and were kept at −80°C until measurement.

The distribution of metabolite concentrations within the tumours was assessed by single photon imaging and quantitative bioluminescence (Mueller-Klieser *et al.*, 1988; Walenta *et al.*, 1990; Mueller-Klieser and Walenta, 1993). For measurement, cryosections made from the frozen tumours were adhered to the upper side of a cover glass. The cover glass was laid upside down upon a glass slide with a rectangular casting mould. The mould was filled with a frozen enzyme solution. The solution contained luciferase and other enzymes that link the substrate of interest to the luciferase light reaction. Different mixtures of enzymes and luciferases had to be used for determination of ATP, glucose, and lactate. Applying these enzyme solutions to serial sections, the metabolites could be determined at quasi-identical locations within the tumours.

The luciferase reaction with light emission started by raising the temperature of the whole array above the melting point by positioning the glass slides into a thermostated chamber on a microscope stage. The spatial distribution of the bioluminescence intensity within the tissue section was measured directly using an appropriate microscope (Axiophot, Zeiss, Oberkochen, Germany) and an imaging photon counting system (ARGUS 100, Hamamatsu, Herrsching, Germany).

After calibration with appropriate standards, two-dimensional density distributions were obtained representing the distribution of ATP, glucose, or lactate in absolute volume-related tissue concentrations ($\mu mol\ g^{-1}$ wet weight). These values were routinely confirmed by independent measurements with high performance liquid chromatography (HPLC) and enzymatic standard assays respectively. The optical overlay of an adjacent tissue section stained with haematoxylin and eosin on the bioluminescence distribution made it possible to evaluate the metabolites in tumour regions with densely packed, viable cancer cells designated 'viable zones'; in areas with necrosis and stromal tissue elements termed 'necrotic zones', in peripheral *vs* central tumour parts; and in adjacent non-tumoric tissue, termed 'normal'. The last tissue type consisted of connective tissue, fat, and skin muscle. Further details on the technique of bioluminescence and imaging photon counting have been published elsewhere (Mueller-Klieser and Walenta, 1993).

For each tumour, up to five single determinations for each substrate were performed and the concentration values were averaged for each histologically classified tissue area. The relative coefficient of variation of these determinations was typically less than 10%; thus, no standard deviation was included for these values.

For further evaluation, those concentration values of single tumours were subsequently averaged for each tumour type and classified tissue area and were given as mean ± s.d. Concentrations in corresponding tissue areas of the two tumour groups were compared by the Mann–Whitney *U*-test, whereas the Wilcoxon test for paired observations was used for the analysis of differences between regions within the tumour sections (e.g. viable *vs* necrotic areas). The dependency of average metabolite concentrations on tumour volume was tested by calculating Spearman's correlation coefficient $r_s$.

### Results

As in previous studies with bioluminescence imaging in various types of tumours, metabolite distributions measured in MF and EE human melanoma xenografts were very heterogeneous and partially reflected the chaotic histological architecture of the malignancies. As mentioned in Materials and methods, the data of all tumours were pooled for each xenograft type and expressed as mean ± s.d. No significant differences between the ATP concentrations averaged over the whole tumour tissue and the adjacent normal tissue were found in the MF and EE xenografts (Figure 1a). Concentration values of glucose were significantly less in the tumour than in the adjacent normal tissue (Figure 1b). In contrast, opposing results were obtained for the distribution of lactate (Figure 1c). Across the two tumour lines mean concentrations of all three metabolites in corresponding tissue areas were very similar. Only ATP values obtained in adjacent normal tissue were significantly different between MF and EE xenografts ($P \leqslant 0.05$).

ATP and glucose concentrations were significantly less in necrotic than in viable tumour areas, whereas lactate concentrations were nearly equal in these tumour parts. This was true in both MF and EE melanomas, as demonstrated in Figure 2a–c. Only for EE xenografts, the lactate concentrations showed a weak significant difference between viable and necrotic tumour parts ($P \leqslant 0.05$). As described above for

**Figure 1** ATP, glucose and lactate concentrations (mean ± s.d.) in whole tumour tissue (■) and in the adjacent normal tissue (□) of MF ($n = 12$) and EE ($n = 13$) human melanoma xenografts. Significant differences between tumour and normal tissue are indicated by stars: **, $P \leqslant 0.01$ and ***, $P \leqslant 0.001$. Significant differences across the two xenografts are indicated by triangle: Δ, $P \leqslant 0.05$. (**a**) ATP, (**b**) glucose, (**c**) lactate.

**Figure 2** ATP, glucose and lactate concentrations (mean ± s.d.) in tumour region with densely packed viable cancer cells (■) and in areas with necrosis, connective and stromal tissue (□) of MF ($n = 12$) and EE ($n = 13$) human melanoma xenografts. Significant differences are indicated by stars: *, $P \leqslant 0.05$ and **, $P \leqslant 0.01$. (**a**) ATP, (**b**) glucose, (**c**) lactate.

values averaged over the entire tumour mass, statistically significant differences between MF and EE tumours were not seen for any of the three metabolites ($P > 0.1$).

The evaluation of metabolite concentrations in peripheral *vs* central tumour areas resulted in data shown in Figure 3. ATP and glucose concentrations were significantly less in central as compared with peripheral zones. No differences were found for lactate, although there was a tendency for higher values in the tumour centre. These findings were obtained in both MF and EE melanomas. Significant differences between the two tumour lines were not registered for any of the metabolites.

Since ATP, glucose and lactate concentrations obtained in the tumour areas showed no significant differences between the two melanoma lines investigated, the values of single tumours from the two entities were pooled for each metabolite and each mode of determination and were investigated for their dependence on tumour size. Figure 4 shows that there was no correlation between ATP, glucose and lactate concentrations obtained in whole tumour areas of single malignancies and tumour volume. As indicated by Spearman's correlation coefficient, no size dependencies were obvious for the concentrations of the three metabolites in viable and necrotic tumour regions or in normal tissue. An example is illustrated in Figure 5 showing ATP concentrations in viable (open symbols) and necrotic (solid symbols) tumour areas as a function of tumour volume.

### Discussion

The results of the present study clearly show that there is no statistically significant or biologically relevant difference in the metabolic micromilieu between the two types of human melanoma xenografts investigated. Obviously, the distribution of ATP, glucose, and lactate reflect the similarity in the vascular density between the two tumour groups rather than the relatively drastic difference in volume growth kinetics. Previous investigations with bioluminescence imaging on hamster melanomas demonstrated that the local tissue concentration of ATP was positively correlated with the regional perfusion (Walenta *et al*., 1992). If one assumes that the morphometric data on the vascularity of the melanomas investigated in this study are representative of the functional state of tumour microcirculation, then similar metabolic microenvironments can in fact be expected in these melanomas.

The conclusions made above are true under the assumption that the efficacy of supply is the key modulator of the metabolic milieu in solid tumours, as it has been demonstrated for several types of human tumour xenografts (Kallinowski *et al*., 1989). In those tumours, high blood flow rates were associated with high metabolic turnover rates and rapid tumour growth. Despite high oxygen consumption rates, the oxygenation status was best in cancers with high-flow rates and fast growth, whereas substantial hypoxia with an oxygen pressure smaller than 5 mmHg was found only in poorly perfused malignancies. The findings of the present investigation suggest that tumours can differ greatly in their growth rate, although their situation with regard to vascular supply and metabolic milieu may be very similar. Obviously, blood supply meets the requirements for the unrestricted expression of intrinsic growth properties in both the tumour entities studied. In such a case, neither vascular parameters, nor data on blood perfusion or the metabolic state of the tumours would allow for a prediction of the rate of tumour expansion. The conclusion is supported by the finding, that EE and

**Figure 3** ATP, glucose and lactate concentrations (mean ± s.d.) in peripheral (■) and central (□) tumour regions of MF ($n = 12$) and EE ($n = 13$) human melanoma xenografts. Significant differences between both regions are indicated by stars: *, $P \leqslant 0.05$, **, $P \leqslant 0.01$ and ***, $P \leqslant 0.001$. (**a**) ATP, (**b**) glucose, (**c**) lactate.

MF cells in monolayer culture have significantly different cell number doubling times of 15 h and 35 h respectively (EK Rofstad *et al.*, unpublished results).

There is a striking difference in the radiobiologically hypoxic fraction of the two melanoma lines that cannot be explained on the basis of the present data. Although oxygen tension values in these tumours are presently unknown, the congruity of vascular density and metabolite distributions in the two melanoma types argues against profound differences in tumour oxygenation. This conclusion is strongly supported by previous investigations comparing tissue oxygenation of various solid tumours as quantified by cryospectrophotometry with intratumoral concentrations of ATP measured with bioluminescence (Mueller-Klieser *et al.*, 1990). ATP was positively correlated with tissue oxygenation status across the different tumour lines in that experimental series. Intermediate hypoxia can probably not explain the apparent discrepancy between radiobiological hypoxia and metabolic milieu in these tumours since recent data have indicated that human melanoma xenografts do not show significant transient perfusion (Tufto and Rofstad, 1995). On the basis of the present data one can assume that the metabolic micromilieu, i.e. the tissue concentration of oxygen and metabolites, is identical in both xenograft types. Therefore, the striking differences in the radiobiologically hypoxic fraction and the volume growth rate cannot be related to the vascular supply and must be explained by differences in the intrinsic properties of both tumours.

The bioluminescence method only allows for the measurement of steady state concentrations of metabolites. Thus, one possible explanation could be an intrinsically different metabolic turnover rate in both tumour types, which cannot be registered with the bioluminescence method. This explanation is in accordance with the volume growth rate *in vivo* and the

**Figure 4** ATP, glucose and lactate concentrations values averaged over whole tumour areas of MF (●) and EE (△) human melanoma xenografts as a function of tumour volume. (**a**) ATP, (**b**) glucose, (**c**) lactate. Each symbol represents the mean concentration value for one tumour obtained from up to five single measurements. Since the relative coefficient of variation of these determinations was typically less than 10%, no standard deviation is included.

**Figure 5** ATP concentrations in viable (open symbols) and necrotic (solid symbols) tumour regions of MF (circles) and EE (triangles) human melanoma xenografts as a function of tumour volume. Each symbol represents the mean concentration value for one tumour obtained from up to five single measurements. Since the relative coefficient of variation of these determinations was typically less than 10%, no standard deviation is included.

cell growth rate *in vitro*. In addition, tumour cells in MF xenografts with lower metabolic turnover rates may survive hypoxic supply conditions for a longer time, leading to a higher radiobiologically hypoxic fraction as compared with EE tumours.

The major advance in using metabolic imaging with bioluminescence as compared with previous approaches is the accessibility of structure-associated information on the functional state of biological tissue. In most cases, the technique allows for a highly resolved distinction between data acquired in malignancies and values measured in surrounding normal tissue. The concentrations of ATP and glucose were significantly lower in necrotic tumour regions with stromal elements as compared with areas with densely packed viable cancer cells, whereas lactate concentrations were equal or showed only a weak difference in these zones. Consequently, the distribution of ATP and glucose mirrored the geometry of these two 'classes' of tumour regions contrasted by lactate distributions that seemed to be relatively unrelated to the tissue architecture. Accordingly, the finding of significantly less ATP and glucose in the tumour centre compared with the periphery may reflect a different proportion of necrotic tissue in outer and inner tumour regions. Preliminary data obtained with the bioluminescence technique in biopsies of cervical cancers are indicative of structure-related patterns of all three metabolites in those malignancies (Mueller-Klieser *et al.*, 1994). Considering the regional evaluation of metabolites, the absence of any significant difference in the global metabolite concentrations between the two melanoma lines is consistent with the determination of similar necrotic fractions in these cancers (Solesvik *et al.*, 1982).

The present study documents no significant changes of the measuring parameters as a function of tumour size. This result may indicate no change in the proportion of necrotic *vs* viable tissue as the tumours increase in volume. On the other hand, a relatively high ATP content in the necrotic region is indicative of some residual metabolic activity in those areas. Investigations on multicellular tumour spheroids have also revealed such a residual metabolic turnover in regions that may be identified as being necrotic by histological criteria (Walenta *et al.*, 1990). Taking into account the absence of correlation, it seems reasonable to calculate averages irrespective of tumour volume for the parameters considered here.

In conclusion, quantitative bioluminescence with single photon imaging offers the possibility of regional and structure-related evaluation of metabolite concentrations in biological tissue. The application of this technique in two lines of human melanoma xenografts with similar vascularity, yet different growth rates and different radiobiologically hypoxic fractions resulted in metabolite distributions that were not statistically different. Thus, the measured metabolite concentrations reflect the efficiency of tumour blood perfusion and do not necessarily predict intrinsic growth behaviour or therapeutic sensitivity of tumours.

**Acknowledgements**

This work was supported by grant 01Z08801 of the Bundesministerium für Forschung und Technologie and The Norwegian Cancer Society

**References**

GATENBY RA, KESSLER HB, ROSENBLUM JS, COIA LR, MOLDOFSKY PJ, HARTZ WH AND BRODER GJ. (1988). Oxygen distribution in squamous cell carcinoma metastases and its relationship to outcome of radiation therapy. *Int. J. Radiat. Oncol. Biol. Phys.*, **14**, 831–838.

HÖCKEL M, SCHLENGER K, KNOOP C AND VAUPEL P. (1991). Oxygenation of carcinomas of the uterine cervix–evaluation by computerized $0_2$ tension measurements. *Cancer Res.*, **51**, 6098–6102.

HÖCKEL M, KNOOP C, SCHLENGER K, VORNDRAN B, BAUSSMANN E, MITZE M, KNAPSTEIN PG AND VAUPEL P. (1993). Intratumoral $pO_2$ predicts survival in advanced cancer of the uterine cervix. *Radiother. Oncol.*, **26**, 45–50.

KALLINOWSKI F, SCHLENGER K, RUNKEL S, KLOES M, STOHRER M, OKUNIEFF P AND VAUPEL P. (1989). Blood flow, metabolism, cellular microenvironment, and growth rate of human tumor xenografts. *Cancer Res.*, **49**, 3759–3764.

KROEGER M, WALENTA S, ROFSTAD EK AND MUELLER-KLIESER W. (1991). Imaging of structure and function in human tumor xenografts. In *Tumour Blood Supply and Metabolic Microenvironment. Funktionsanalyse biologischer Systeme*. **20**, Vaupel P, Jain RK. (eds) pp. 305–318. Gustav Fischer: Stuttgart.

KUHNLE GEH, DELLIAN M, WALENTA S, MUELLER-KLIESER W AND GOETZ AE. (1992). Simultaneous high-resolution measurement of adenosine triphosphate levels and blood flow in the hamster amelanotic melanoma A-Mel-3. *J. Natl Cancer Inst.*, **84**, 1642–1647.

MUELLER-KLIESER W AND WALENTA S. (1993). Geographical mapping of metabolites in biological tissue with quantitative bioluminescence and single photon imaging (Invited review). *Histochem J.*, **24**, 407–420.

MUELLER-KLIESER W, WALENTA S, PASCHEN W, KALLINOWSKI F AND VAUPEL P. (1988). Metabolic imaging in microregions of tumors and normal tissues with bioluminescence and photon counting. *J. Natl Cancer Inst.*, **80**, 842–848.

MUELLER-KLIESER W, SCHAEFER C, WALENTA S, ROFSTAD EK, FENTON BM AND SUTHERLAND RM. (1990). Assessment of tumor energy and oxygenation status by bioluminescence, nuclear magnetic resonance spectroscopy, and cryospectrophotometry. *Cancer Res.*, **50**, 1681–1685.

MUELLER-KLIESER W, WALENTA S AND SCHWICKERT G. (1994). Quantitative bioluminescence imaging–a method for the detection of metabolite distributions in frozen tissues. *SPIE Proc.*, **2083**, 34–40.

ROFSTAD EK. (1984). Growth and vascular structure of human melanoma xenografts. *Cell Tissue Kinet.*, **17**, 91–101.

ROFSTAD EK. (1989*a*). Local tumor control following single dose irradiation of human melanoma xenografts: relationship to cellular radiosensitivity and influence of an immune response by the athymic mouse. *Cancer Res.*, **49**, 3163–3167.

ROFSTAD EK. (1989*b*). Hypoxia and reoxygenation in human melanoma xenografts. *Int. J. Radiat. Oncol. Biol. Phys.*, **17**, 81–89.

ROFSTAD EK AND BRUSTAD T. (1981). Radiation response *in vitro* of cells from five human malignant melanoma xenografts. *Int. J. Radiat. Biol.*, **40**, 677–680.

ROFSTAD EK AND BRUSTAD T. (1986). Arrhenius analysis of the heat response *in vivo* and *in vitro* of human melanoma xenografts. *Int. J. Hyperthermia*, **2**, 359–368.

SCHAEFER C, MAYER WK, KRÜGER W AND VAUPEL P. (1993). Microregional distributions of glucose, lactate, ATP and tissue pH in experimental tumours upon local hyperthermia and/or hyperglycaemia. *J. Cancer Res. Clin. Oncol.*, **119**, 599–608.

SOLESVIK OV, ROFSTAD EK AND BRUSTAD T. (1982). Vascular structure of five human malignant melanomas grown in athymic nude mice. *Br. J. Cancer*, **46**, 557–567.

SOLESVIK OV, ROFSTAD ED AND BRUSTAD T. (1984). Vascular changes in a human malignant melanoma xenograft following single-dose irradiation. *Radiat. Res.*, **98**, 115–128.

TUFTO I AND ROFSTAD EK. (1995). Transient perfusion in human melanoma xenografts. *Br. J. Cancer*, **71**, 789–793.

VAUPEL P, KALLINOWSKI F AND OKUNIEFF P. (1989). Blood flow, oxygen and nutrient supply, and metabolic microenvironment of human tumors: a review. *Cancer Res.*, **49**, 6449–6465.

VAUPEL P, SCHLENGER K, KNOOP C AND HÖCKEL M. (1991). Oxygenation of human tumors: evaluation of tissue oxygen distribution in breast cancers by computerized $O_2$ tension measurements. *Cancer Res.*, **51**, 3316–3322.

WALENTA S, DOETSCH J AND MUELLER-KLIESER W. (1990). ATP concentrations in multicellular tumor spheroids assessed by single photon imaging and quantitative bioluminescence. *Eur. J. Cell Biol.*, **52**, 389–393.

WALENTA S, DELLIAN M, GOETZ AE, KUHNLE GEH AND MUELLER-KLIESER W. (1992). Pixel-to-pixel correlation between images of absolute ATP concentrations and blood flow in tumours. *Br. J. Cancer.*, **66**, 1099–1102.